# A new treatment strategy for end-stage hepatic alveolar echinococcosis: IVC resection without reconstruction

**DOI:** 10.1038/s41598-019-45968-5

**Published:** 2019-07-01

**Authors:** Qiancheng Du, Yanyan Wang, Mengzhao Zhang, Yichong Chen, Xuepeng Mei, Yanfei Li, Ying Zhou, Haining Fan

**Affiliations:** 1 Department of General Surgery, Shanghai Fourth People’s Hospital Affiliated to Tongji University School of Medicine, Shanghai, 200081 China; 2 The Graduate School, Qinghai University, Xining, 810000 China; 3 Department of Hematology, Affiliated Fuyang Hospital of Anhui Medical University, Fuyang, 236000 China; 4 Department of Hepatopancreatobiliary Surgery, the Affiliated Hospital of Qinghai University and Qinghai Province Key Laboratory of Hydatid Disease Research, Qinghai University, Xining, 81000 China

**Keywords:** Parasitic infection, Reconstruction, Parasitic liver diseases

## Abstract

Patients with hepatic alveolar echinococcosis (HAE) infringing on the inferior vena cava (IVC) have a poor prognosis when radical resection cannot be performed because curative resection is limited by IVC reconstruction. There is little information concerning combined resection of the liver and the IVC. This study explored a novel treatment method for HAE infringing on the IVC and evaluated the safety and feasibility of combined resection of the liver and the IVC. A total of 13 patients were treated with liver resection combined with IVC resection for end-stage HAE between January 2016 and July 2018 at the Affiliated Hospital of Qinghai University. The demographic, clinical, and follow-up data were collected and analysed. The 13 patients underwent resection of the IVC without reconstruction. Of these, 3 exhibited oedema of both lower limbs and the scrotum (23.1%), 2 exhibited pneumothorax (15.4%), 1 exhibited bile leakage (7.7%), 1 exhibited bacteraemia (7.7%), and 1 developed abdominal haemorrhage that was stopped with conservative treatment (7.7%). There was 1 case of operation-related mortality because of upper gastrointestinal haemorrhage (7.7%), and no patients developed recurrence or had residual lesions. Liver resection combined with IVC resection is effective and feasible for patients with HAE infringing on the IVC.

## Introduction

Humans are occasionally infected by the metacestodes of *Echinococcus multilocularis* (EM), largely because of travel and domestic dogs. These metacestodes are predominately located in the liver (60–70%), while a few can be located in the lungs, other abdominal organs, the brain, osseous tissue, and even the thyroid^1^. Approximately 70% of HAE lesions are in the right lobe of the liver, with 40% encroaching on the hepatic hilus and rarely on the IVC^2^. In the liver, the larval lesion grows by exogenous germination, and the liver is impaired by direct erosion, compression, and an inflammatory reaction to EM, which may finally result in liver failure and possibly severe or life-threatening complications, including obstructive jaundice, ascites, and portal hypertension associated with severe cirrhosis^3^. The diagnosis of alveolar echinococcosis (AE) mainly depends on the following clues: epidemiological evidence (contact history in endemic area), signs and symptoms, serological findings (human echinococcus IgG antibody by ELISA), imaging findings (computed tomography [CT], magnetic resonance imaging [MRI], ultrasound), intraoperative probing and postoperative pathological diagnosis^4^. AE is also difficult to find at an early stage, and the lesions are typically large, multiple, or even infringing on adjacent structures at the first diagnosis. Indeed, AE presents a malignant-like behaviour, and approximately 90% of patients with advanced-stage tumours in whom radical resection cannot be performed will die within 10 years of being diagnosed^5^. The prognosis of AE is not optimistic. From 1960 to 1972, the mortality rate was 70% and 94% after 5 and 10 years, respectively^4^. Surgery is the first-choice treatment method whenever feasible, but radical surgery is only possible in 35% of patients, and is especially difficult when the first and second hepatic portal veins are involved^6^. IVC reconstruction may be required in some complex surgeries, such as surgeries for end-stage cancer (hepatocellular carcinoma), advanced HAE and major trauma to the IVC; however, IVC reconstruction still does not achieve satisfactory results and increases the potential risk of infection, bleeding, vascular stenosis and thrombogenesis^7,8^. It has been reported that artificial blood vessels can be used to reconstruct the IVC after resection, but various complications can occur during different periods after the surgery^9^. Although liver resection combined with IVC resection has a high surgical risk, curative surgical resection could improve the prognosis and long-term survival of patients with IVC infringement. Recent advances in techniques for vascular surgery and hepatic surgery, such as total hepatic vascular exclusion, have extended the surgical indications for such end-stage tumours^10^, but it still remains a controversy whether the IVC should be reconstructed. In the present study, given the high incidence of complications after reconstruction of the IVC, we present our experience using liver resection combined with IVC resection in the treatment of HAE infringing on the IVC.

## Results

### Patient characteristics

We collected data from 13 patients with HAE infringing on the IVC who underwent liver resection combined with IVC resection during the study period and who met the common requirements of the surgery team. None of the 13 patients received oral albendazole before surgery because all patients were first diagnosed at the time of surgery. The mean (SD) age of the patients was 34.6 (10.5) years, with an age range of 15–49 years. The mean (SD) follow-up time was 23.7 (10.6) months, ranging from 13 to 45 months. The baseline characteristics of the included patients with HAE infringing on the IVC are listed in Table 1.

**Table 1:** Baseline characteristics of included patients with HAE infringing on the IVC.

No.	Sex	Age (year)	ALT (U/L)	AST (U/L)	ALP (U/L)	GGT (U/L)	CHE (U/L)	TBIL (µmol/L)	DBIL (µmol/L)
1	Female	28	13.5	74.4	118	20	4645	4.9	0.4
2	Female	33	40	57	149	52	2678	7	5.1
3	Female	35	35	31	284.2	369.6	4998	10	4.7
4	Female	42	53	58	683.3	170.8	2891	87.4	82.5
5	Male	43	19	23	99.9	36.3	5586	13.1	8.7
6	Female	40	19	24	241	204	7096	42	24.5
7	Female	18	13	21	101	14	8984	6.2	2
8	Male	47	35	25	557	158	3510	33	16.3
9	Male	49	24	31	92.5	50	2987	21	14.1
10	Male	32	10	23	371	148	1824	34	14.7
11	Female	27	10	20	67	10	5922	8.9	1.7
12	Male	41	21	20	142	47	3674	14.7	7.7
13	Male	15	38	36	138	45	3842	9.5	4.5

Abbreviations, ALT: alanine transaminase, AST: aspartate amino transferase, ALP: alkaline phosphatase, GGT: gamma-glutamyl transpeptidase, CHE: cholinesterase, TBIL: total bilirubin, DBIL: direct bilirubin.

### Imaging characteristics

**Figure 1:**
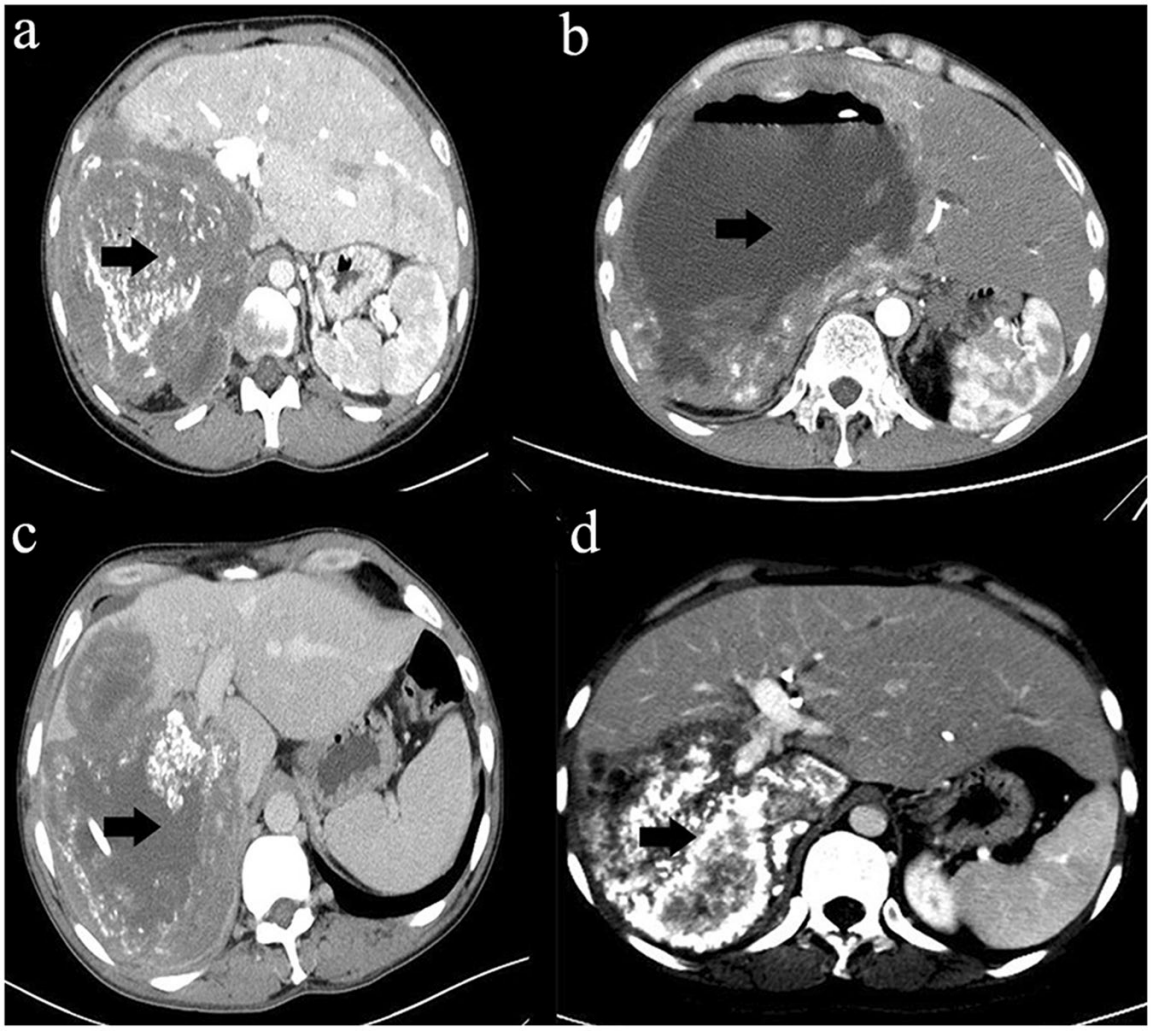
Abdominal images. The black arrows show the HAE lesion.

The common symptom on admission in all patients was nonspecific upper abdominal pain; 3 cases were found by physical examination. CT or MRI showed multiple, large, irregular space-occupying lesions (Fig. 1), and the blood of all patients was positive for hydatid antibodies. All patients were diagnosed with HAE infringing on the IVC, and the inner diameter of the IVC was completely blocked (in one of the patients, 4/5 of the inner diameter had already been violated). According to the PNM classification (Table 2)^11^, 2 patients were P4N0M0, 6 patients were P4N1M0, and 5 patients were P4N1M1. Extrahepatic lesions were located in the lungs in 4 cases (4/13), were located in the brain in 2 cases (2/13), infringed on the diaphragm in 6 cases (6/13), and infringed on the right adrenal gland in 6 cases (6/13). The main lesion in 12 patients was in the right lobe of the liver, and the main lesion in 1 patient was in the left lobe of the liver. The right hepatic vein and middle hepatic vein were invaded in 9 patients, the right hepatic vein was invaded in 2 patients, the left hepatic vein was invaded in 1 patient, and all three veins were invaded in 1 patient. The trunk of the portal vein was invaded in 4 patients, the left portal vein was invaded in 1 patient, and only the right portal vein was invaded in 7 patients. The mean maximum cross-sectional area of the lesion was 141.73 ± 56.37 cm^2^, ranging from 45.07 to 262.72 cm^2^ (Table 3).

**Table 2:** PNM classification of alveolar echinococcosis.

	WTO PNM classification P_0–4_ N_0–1_ M_0–1_
P	Hepatic localization of the parasite
P X	Primary tumour cannot be assessed
P 0	No detectable tumour in the liver
P 1	Peripheral lesions without proximal vascular and/or biliary involvement
P 2	Central lesions with proximal vascular and/or biliary involvement of one lobe^a^
P 3	Central lesions with hilar vascular or biliary involvement of both lobes and/or with involvement of two hepatic veins
P 4	Any liver lesion extending along the vessels^b^ and the biliary tree
N	Extrahepatic involvement of neighbouring organs [diaphragm, lungs, pleura, pericardium, heart, gastric and duodenal wall, adrenal glands, peritoneum, retroperitoneum, parietal wall (muscles, skin, bone), pancreas, regional lymph nodes, liver ligaments, kidneys]
N X	Not evaluable
N 0	No regional involvement
N 1	Regional involvement of contiguous organs or tissues
M	Absence or presence of distant metastasis [lungs, distant lymph nodes, spleen, CNS, orbitals, bone, skin, muscles, kidneys, distant peritoneum and retroperitoneum]
M X	Not completely evaluated
M 0	No metastasis^c^
M 1	Metastasis

^a^For classification, the plane projecting between the bed of the gall bladder and the inferior vena cava divides the liver into two lobes. ^b^Vessels refers to the inferior vena cava, portal vein and arteries. ^c^Negative chest X-ray and cerebral CT.

**Table 3:** Imaging characteristics of the included patients with HAE.

No.	No. of lesions	Cross-sectional area of largest lesion	Lesion location	Extrahepatic lesion	Complete IVC blockage	Collateral circulation formation
1	1	157.54	S4 5 6 7 8	No	Yes	Yes
2	1	117.99	S4 5 6 7 8	Lung	Yes	Yes
3	1	139.35	S1 4 5 6 7 8	Lung	Yes	Yes
4	1	217.76	S4 5 6 7 8	No	Yes	Yes
5	1	107.85	S1 2 3 8	No	No	Yes
6	1	103.58	S1 4 5 6 7 8	No	Yes	Yes
7	1	45.07	S4 5 6 7 8	No	Yes	Yes
8	1	262.72	S4 5 6 7 8	Brain	Yes	Yes
9	>3	126.28	S5 6 7 8	Brain, lung	Yes	Yes
10	1	106.91	S5 6 7 8	No	Yes	Yes
11	1	107.39	S1 4 5 6 7 8	No	Yes	Yes
12	1	182	S5 6 7 8	No	Yes	Yes
13	1	168	S4 5 6 7 8	Lung	Yes	Yes

Abbreviation: S, segment; No., number.

### Treatment

Thirteen patients underwent resection of the IVC without reconstruction, and radical resection was achieved in all patients. Preoperative percutaneous transhepatic cholangial drainage (PTCD) was performed in 2 patients with liquefactive necrosis of the lesions, and 2 patients were treated with PTCD and endoscopic retrograde cholangiopancreatography (ERCP) for obstructive jaundice. Eight patients underwent hepatic right trilobectomy, 3 underwent right hemihepatectomy, 1 underwent left hemihepatectomy, and 1 underwent extended right trilobectomy. Eight patients underwent *ex vivo* liver resection and autologous liver transplantation. During the anhepatic time in these 8 cases, we usually used a portal-caval shunt for haemodynamic maintenance. The mean (SD) length of hospitalization was 49.8 (27.2) days, ranging from 11 to 102 days.

### Postoperative observation

Postoperative complications were defined as complications occurring within 30 days after surgery. After surgery, 3 patients developed oedema of both lower limbs and the scrotum (23.1%), 2 developed pneumothorax (15.4%), 1 patient developed bile leakage (7.7%), 1 patient developed bacteraemia that was cured with corresponding treatment (7.7%), and 1 patient developed abdominal haemorrhage that was stopped with conservative treatment (7.7%). The Clavien–Dindo classification relies on the principle of grading complications based on the therapy used to treat the complication and is divided into 5 levels^12^. Grade I complications were recorded in 2 patients (15.4%), grade II in 10 (76.9%) and grade IIIa in 1 (7.7%).

### Analysis of patients treated with standard resection and ex vivo resection

Among the 13 patients, 5 patients underwent standard resection, and 8 patients underwent *ex vivo* resection. In the standard resection group, there were 4 P4N1M0 cases and 1 P4N1M1 case, significantly more than in the *ex vivo* resection group. The cross-sectional area of the largest lesions in the *ex vivo* resection group (161.25 cm^2^) was greater than that in the standard resection group (110.49 cm^2^). However, the mean length of hospitalization in the standard resection group (59.80 days) remained longer than in that the *ex vivo* resection group (43.50 days). All patients in the *ex vivo* resection group were classified as greater than or equal to grade II according to the Clavien–Dindo classification system. All patients who developed complications during hospitalization were in the *ex vivo* resection group (Table 4).

**Table 4:** Preoperative and postoperative baseline characteristics of patients treated with standard resection and *ex vivo* resection.

Variable	Total	Standard resection	*Ex vivo* resection
**Sex**			
Male	6 (46.2%)	1	5
Female	7 (53.8%)	4	3
^*^ **Symptoms**			
Present	10 (76.9%)	3	7
Absence	3 (23.1%)	2	1
**PNM classification**			
P4N0M0	2 (15.4%)	1	0
P4N1M0	6 (46.1%)	3	4
P4N1M1	5 (38.5%)	1	4
**Clavien–Dindo classification**			
Grade I	2 (15.4%)	2	0
Grade II	10 (76.9%)	3	7
Grade IIIa	1 (7.7%)	0	1
**Complications during hospitalization**			
Oedema of both lower limbs and scrotum	3 (23.1%)	0	3
Pneumothorax	2 (15.4%)	0	2
Bile leakage	1 (7.7%)	0	1
Bacteraemia	1 (7.7%)	0	1
Abdominal haemorrhage	1 (7.7%)	0	1
Cross-sectional area of largest lesion	141.73 ± 56.37	110.49 ± 42.97	161.25 ± 57.09
Age (year)	34.62 ± 10.52	30.60 ± 8.30	37.13 ± 11.47
^*^ALT (U/L)	25.42 ± 13.51	18.10 ± 9.99	30.00 ± 13.92
^*^AST (U/L)	25.0 (22.0–46.5)	24.0 (22.0–52.7)	28.0 (20.8–51.8)
^*^ALP (U/L)	142.0 (100.5–327.6)	241.0 (109.5–327.6)	140.0 (94.4–455.0)
^*^GGT (U/L)	50.0 (28.2–164.4)	148.0 (17.0–286.8)	48.5 (38.5–131.5)
^*^CHE (U/L)	4510.54 ± 1995.17	5509.40 ± 2700.97	3886.25 ± 1223.64
^*^TBIL (µmol/L)	13.1 (8.0–33.5)	10.0 (5.6–38.0)	13.9 (9.1–30.0)
^*^DBIL (µmol/L)	7.7 (3.3–15.5)	4.7 (1.2–19.6)	8.2 (4.7–15.8)
Follow-up time	18.0 (15.5–35.0)	18.0 (17.0–35.0)	20.0 (13.5–33.0)
Length of hospitalization	49.77 ± 27.19	59.80 ± 23.70	43.50 ± 28.81

*All refers to preoperative indicators.

### Patient follow-up

With a median follow-up duration of 23.7 months, ranging from 13 to 45 months, all patients returned to the outpatient clinic and received regular follow-up examinations after approximately 6 months and 12 months. At approximately 6 months, all patients had normal liver function, except 1 patient with mildly elevated enzyme levels and 1 patient suffering from persistent malnutrition with long-term nutritional strengthening. Examination by abdominal CT showed that the residual liver compensated well in all patients. One patient showed non-specific gastrointestinal symptoms, while the other patients showed no symptoms of discomfort. At approximately 6 months, the patient with preexisting discomfort showed no significant improvement in the symptoms and presented with cough, nausea and other discomfort. Zero out of the 13 patients experienced recurrence or had residual lesions. There was 1 case of operation-related mortality because of upper gastrointestinal haemorrhage (Table 5).

**Table 5:** Basic characteristics of the patients during the follow-up period.

NO.	6 months				12 months				Prognosis
	Liver function	Complications	Liver compensatory function	Symptoms	Liver function	Complications	Liver compensatory function	Symptoms	
1	normal	No	normal	No	normal	No	normal	No	Survival
2	normal	No	normal	No	normal	No	normal	No	Survival
3	normal	No	normal	No	normal	No	normal	No	Survival
4	normal	No	normal	No	normal	No	normal	No	Survival
5	normal	No	normal	No	normal	No	normal	No	Survival
6	normal	No	normal	No	normal	No	normal	No	Survival
7	normal	No	normal	No	normal	No	normal	No	Survival
8	mildly abnormal	malnutrition	normal	Yes	mildly abnormal	malnutrition URI	mildly abnormal	Yes	Death
9	normal	No	normal	No	normal	No	normal	No	Survival
10	normal	No	normal	No	normal	No	normal	No	Survival
11	normal	No	normal	No	normal	No	normal	No	Survival
12	normal	No	normal	No	normal	No	normal	No	Survival
13	normal	No	normal	No	normal	No	normal	No	Survival

Abbreviations, URI: upper respiratory infection.

## Discussion

The treatment of end-stage HAE is complicated and can range from drug treatment to radical resection. Surgical therapy still remains the first-line treatment for end-stage HAE^13,14^. Brain metastasis is not a contraindication to surgery, and patients whose metastases were not located in important areas of the brain could still be treated with surgery. Previous treatments for HAE patients with IVC involvement include drug therapy, palliative treatment [PTCD, ERCP+ endoscopic retrograde biliary drainage (ERBD) and palliative surgery] and radical surgery^13,15^. In our study, all patients with HAE infringing on the IVC underwent liver resection combined with IVC resection, and this treatment has been reported in a few cases to be a safe and effective surgical scheme in end-stage HAE^16^. In comparison with IVC reconstruction after resection, the merits of resecting the IVC without reconstructing it are obvious and include a shorter operative time, less operative trauma, less anticoagulant drug use, and fewer complications^17^. Furthermore, compared with palliative resection, our approach resulted in no postoperative recurrence or residual lesions, which might indicate that radical resection can essentially be achieved by liver resection combined with IVC resection.

The studies investigating resection of the IVC are relatively rare. Unlike previously published case reports^8^, our case series includes patients with complex liver lesions. This series describing 13 patients who underwent IVC resection for HAE infringing on the IVC without prosthetic or autologous reconstruction is the largest to date. IVC involvement was previously an indication for palliative resection. Christian Partensky reported the cases of 18 patients who required liver resection for HAE. Of 9 patients who underwent palliative resection, 2 died during the follow-up period, indicating that IVC involvement does not have a survival advantage for HAE patients^18^. Unlike their results, our retrospective study of HAE infringing on the IVC, with a median follow-up duration of 18 months, showed that there was 1 case of operation-related mortality. Our team concluded that the cause of death was complex hepatectomy and that it had nothing to do with the absence of the IVC. These outcomes highlight the important opinion that patients undergoing IVC resection could achieve a radical cure and that less operative trauma could reduce the rate of surgery-related mortality.

Biliary leakage is a common complication of liver resection that led to persistent fever in 1 patient in our study (7.7%). The patient was cured after reducing the pressure in the biliary tract by endoscopic nasobiliary drainage (ENBD). Dziri *et al*.^19^ reported that omentoplasty could reduce the occurrence rate of postoperative biliary leakage after surgery for hepatic echinococcosis in a prospective, multi-centre, randomized trial. Ijichi *et al*.^20^ reported that the biliary leakage test was not an effective means of detecting biliary leakage in a randomized controlled study of 103 patients treated with hepatic resection. Our results show that bile leakage mainly occurred in the *ex vivo* resection group, which might be related to surgical trauma. Therefore, radical surgery should be performed as much as possible, and the surgeon should minimize unnecessary cutting of the liver surface area.

**Figure 2:**
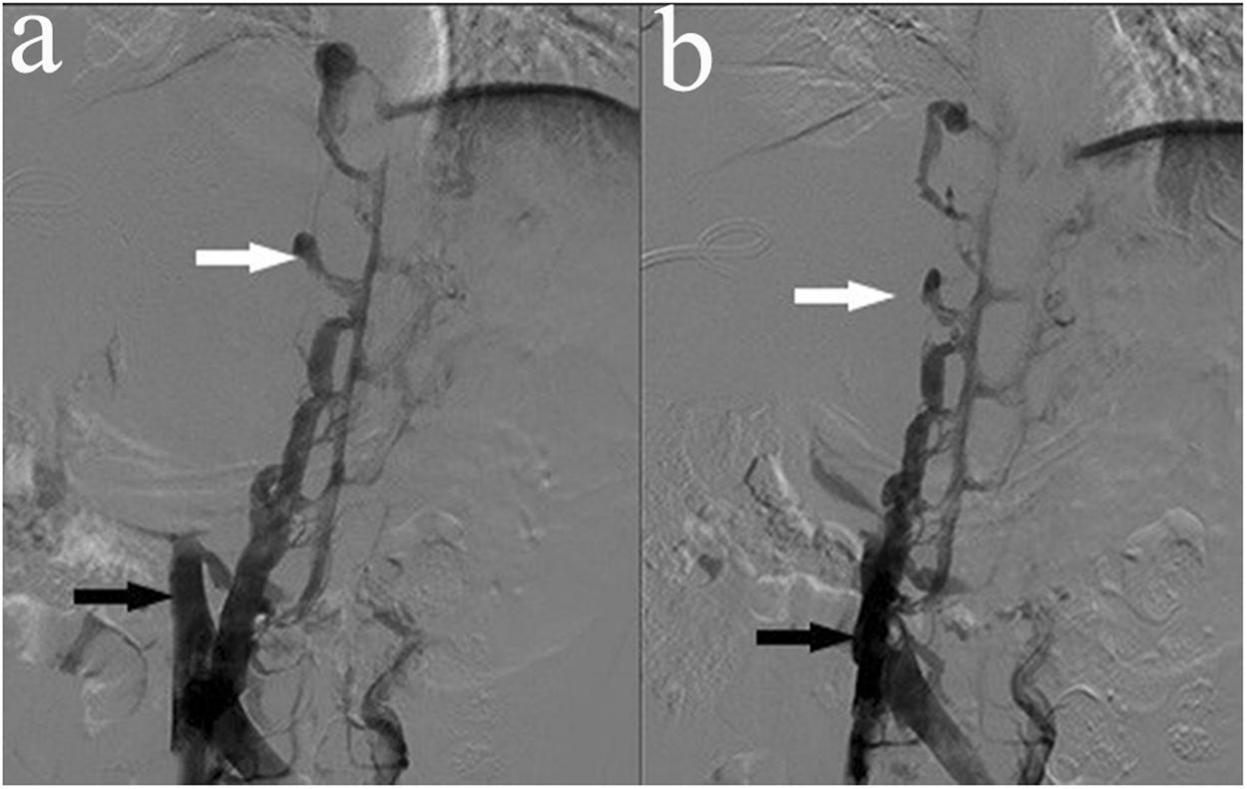
Inferior vena cava angiographs. The white arrows show the compensatory azygos vein, and the black arrows show the obstructed IVC.

In patients with complete IVC blockage, the collateral circulation could provide sufficient venous return, and therefore, corresponding symptoms, such as lower limb and scrotal oedema, are uncommon. In this study, the IVC was completely blocked in 12 patients with HAE infringing on the IVC, and 4/5 of the inner diameter was violated in 1 patient. In our cases, preoperative IVC venography showed suitable compensation by the collateral circulation in all patients (Fig. 2). Hence, IVC reestablishment may not be feasible in these patients. In line with previous results^21^, no patients showed lower limb or scrotal oedema before the operation. However, in our study, 3 patients had lower limb and scrotal oedema after combined resection of the liver and RHIVC. All of these patients were in the *ex vivo* resection group. Therefore, it could not be determined whether the complication was related to the surgical trauma or the surgical approach. Further study is needed to elucidate whether it this complication is related to removal of the RHIVC. We believe that IVC reestablishment might be beneficial in patients who have poor compensation via the collateral circulation preoperatively.

Infection and thrombosis are the 2 major complications of IVC reconstruction, but both are uncommon. There may not be any clinical means of decreasing the incidence of graft thrombosis aside from short-term anticoagulation treatment^22^. In our study, all patients underwent IVC resection without reconstruction, and anticoagulation therapy was rarely used after surgery. The literature shows that the^23^ operation-related mortality rate of liver resection combined with IVC resection and artificial vascular graft reconstruction is 8.1%. To the best of our knowledge, there have been no studies strictly comparing the surgical outcomes of combined resection of the liver and the IVC with and without IVC reconstruction. Postoperative haemorrhage, including abdominal or gastrointestinal haemorrhage, is a common factor affecting mortality. From 1960 to 1972, the mortality rate was 70% and 94% after 5 and 10 years, respectively^4^. However, in our study, there was 1 case of operation-related mortality due to upper gastrointestinal haemorrhage (7.7%), which is a relatively low rate compared to those previously reported. It is possible that gastrointestinal bleeding might be a reason for portal venous system reconstruction because the patient was in the *ex vivo* resection group.

Renal dysfunction is another complication of performing IVC resection. It has been reported that^21^ patients with obstruction of the suprarenal IVC and a poor collateral circulation are at an increased risk of renal failure after IVC ligation. In our series, 1 patient had transient renal insufficiency after removal of the IVC and was cured with the corresponding treatment. The postoperative serum creatinine level was increased continuously for a short period of time in this patient who did not undergo IVC reconstruction. We believe that the reason for this outcome might be systemic postoperative infection. Hardwigsen *et al*.^8^ proposed that IVC reconstruction might be indispensable if haemodynamics are unstable intraoperatively or postoperatively. In our study, all patients underwent hepatectomy, and it was common to encounter unstable haemodynamics while blocking the hepatic portal system or during extracorporeal circulation. However, none of our patients developed operation-related renal failure. We also determined the Clavien–Dindo classification of the 13 patients; only 1 patient was classified as grade IIIa according to the postoperative therapy for patients with complicated hepatectomy because the patient needed ERCP and endoscopic nasobiliary drainage (ENBD) to reduce the biliary tract pressure, which might have been due to biliary reconstruction during autologous liver transplantation. Therefore, these complications were not the result of removal of the IVC.

This study was the first to explore the performance of IVC resection without reconstruction in treating end-stage hepatic alveolar echinococcosis. Furthermore, the preoperative preparation in these cases was performed well, the operations were difficult, and the surgical approach was unique. Additionally, this article indirectly reflects the shortcomings of previous reports and could serve as a reference for removal of the inferior vena cava. The extent of inferior vena cava invasion was used as an index for the surgical method. This method avoids residual lesions and could achieve radical resection to treat patients with multiple involved organs. The study also has some limitations. First, for studying this type of surgery, a larger sample size should be used. Second, this surgical method also lacks evidence from clinical prospective studies and multi-centre collaboration to verify the findings. Third, the study could not completely eliminate the effect of different types of hepatectomy.

## Conclusion

Liver resection combined with IVC resection is effective and feasible in patients with HAE infringing on the IVC.

## Methods

This study was approved by the Institutional Research Ethics Board of Qinghai University Affiliated Hospital and conformed to the Declaration of Helsinki (No. P-SL-2018005). The requirement of written informed consent was waived because of the retrospective nature of the study. This study did not involve human or animal tissue or blood samples.

### Study design

We retrospectively collected information related to patients treated for HAE infringing on the IVC by liver resection combined with IVC resection at the Affiliated Hospital of Qinghai University, Qinghai, China, from January 2016 to July 2018. The demographic, clinical, and follow-up data were collected and analysed.

### Patients

**Figure 3:**
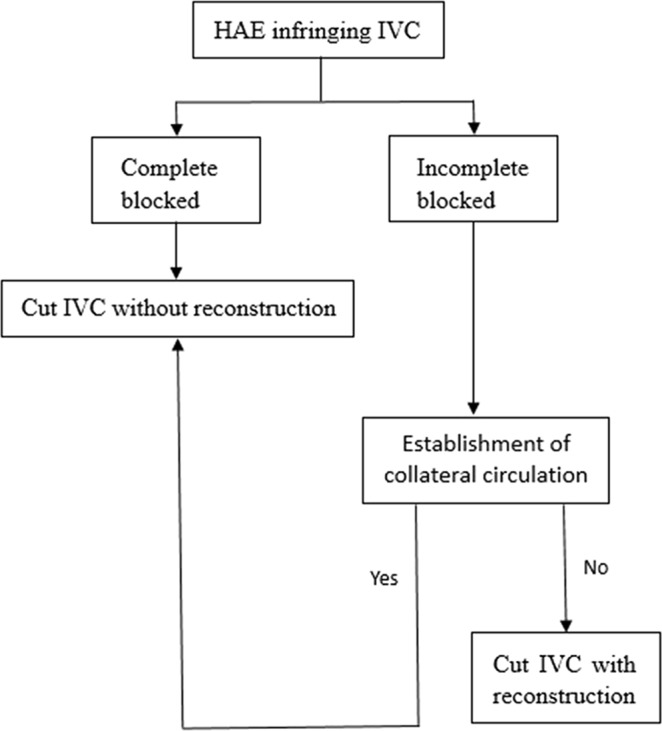
A simplified flow diagram summarizing the selection of the surgical procedure.

Patients were selected according to the common protocol of our team: diagnosis of HAE infringing on the IVC; age <50 years; patients who could undergo radical resection; future remnant liver volume (FRLV) >30%; patients with Child–Pugh classification A or B who could reach A with appropriate intervention; no operative history; no serious concomitant diseases; and indocyanine green retention rate at 15 minutes (ICG R15) <10%. Patients with hepatocellular jaundice, hepatitis B virus DNA quantity >10^4^, patients with any organ failure, patients with abnormal heart or pulmonary function, patients with haematological diseases and patients who could not tolerate general anaesthesia or tracheal intubation were excluded according to our team’s experience. All patients who were selected consented to operative treatment. Figure 3 presents a simplified flow diagram.

### Clinical data

Baseline data, including sex, age, symptom, location of lesion, size of lesion, involvement of blood vessels and bile ducts, operative method, Child–Pugh classification, and postoperative complications, were collected. Characteristics of patients during the follow-up period were recorded, including the results of CT or MRI. End-stage HAE was defined as a lesion invading most of the liver and important vessels, induces obstructive jaundice and portal hypertension, among other conditions, and leads to the inability to perform radical liver resection^5,11,14,24^. Recurrence of HAE was defined as AE occurring at the primary lesion site in the liver after radical resection. Radical resection of HAE was defined as complete removal of the lesion through surgery.

### Surgery

An accurate evaluation was necessary before each operation, with the aim of reducing the risk of serious complications, such as liver failure. We usually used PTCD or biliary stent placement by ERCP in patients with obstructive jaundice to achieve reduce the jaundice preoperatively. To further ensure the safety of each operation, we also performed three-dimensional (3D) reconstruction of the liver and virtual excision of the liver using a computer software system before the operation (IQQA®-Liver, EDDA technology, USA). The indocyanine green retention rate at 15 minutes (ICG R15) needed to be <10% for each patient 1 week preoperatively.

An abdominal inverse L-shaped incision or J-shaped incision was performed for a complicated operation to achieve excellent exposure of the abdominal cavity after general anaesthesia was induced. After careful surgical exploration, we performed hepatectomy or total *ex vivo* autologous liver transplantation for advanced HAE according to the preoperative 3D reconstruction and virtual excision of the liver. We also had to perform a stepwise resection because of the large lesion and limited operating space. We found that the RHIVC was infiltrated and that its inner diameter was completely blocked (in one of these patients, 4/5 of the inner diameter had already been violated). Given that there is still no ideal graft material, reconstruction of the RHIVC could cause greater trauma and increase the risk of thrombosis. Therefore, we decided to remove the IVC. To perform *ex vivo* liver resection and autologous liver transplantation, we ligated and cut the blocked IVC between the upward side of the confluence of the three hepatic veins and 1 cm above the confluence of the left renal vein. For patients only undergoing hepatectomy, we usually ligated and cut the blocked IVC between 1 cm below the confluence of the hepatic vein and 1 cm above the confluence of the left renal vein.

The surgeon and the anaesthesiologist jointly conducted a cardiopulmonary function assessment of the patient, including real-time cardiography, blood pressure monitoring, fingertip oximetry, real-time central venous pressure monitoring, arterial blood gas analysis and intraoperative ultrasound for approximately 20 minutes after the RHIVC was removed, and then we confirmed that the patient had normal respiratory and circulatory function.

### Postoperative management and follow-up

All patients underwent long-term treatment with oral albendazole after the surgery, especially patients brain and lung metastases (15 mg/kg/day for at least 6–12 months)^25^. Routine blood and liver function tests were performed at least every 6 months during medical treatment. During the follow-up period, CT or MRI and liver function tests were performed at 6 and 12 months and then every year after the operation. We used the Clavien–Dindo classification system to assess short-term postoperative complications.

### Statistical analysis

Statistical analysis of the data was performed using SPSS software version 23.0 (IBM Corporation, 2015, USA). Normally distributed continuous variables are described as the mean ± standard deviation, and variables without a normal distribution are described as the median. Qualitative variables are presented as numbers and proportions.

## Data Availability

The datasets generated during and/or analysed during the current study are available from the corresponding author on reasonable request.

## References

[1] Piarroux M (2011). Clinical features and evolution of alveolar echinococcosis in France from 1982 to 2007: results of a survey in 387 patients. J. Hepatol..

[2] Yang X (2017). Risk factors and a simple model for predicting bile leakage after radical hepatectomy in patients with hepatic alveolar echinococcosis. Medicine.

[3] Graeter T (2015). Hepatobiliary complications of alveolar echinococcosis: a long-term follow-up study. World J. Gastroenterol..

[4] Pawlowski, Z. *et al*. Echinococcosis in humans: clinical aspects, diagnosis and treatment in *WHO/OIE manual on echinococcosis in humans and animals: a public health problem of global concern* (eds De, F. *et al*.) 20–72 (World Organisation for Animal Health, 2001).

[5] Gottstein B (1992). Molecular and immunological diagnosis of echinococcosis. Clin. Microbiol. Rev..

[6] Bressonhadni S (2000). A twenty-year history of alveolar echinococcosis: analysis of a series of 117 patients from eastern France. Eur. J. Gastroen. Hep..

[7] Dijana J, Reddy PP, Flynn LM, Robert P (2005). A single center experience in the use of polyurethaneurea arteriovenous grafts. Nephrol. News Issues.

[8] Hardwigsen J (2001). Resection of the inferior vena cava for neoplasms with or without prosthetic replacement: a 14-patient series. Ann. Surg..

[9] Hu H (2017). Liver autotransplantation and retrohepatic vena cava reconstruction for alveolar echinococcosis. J. Surg. Res..

[10] Miyazaki M (1999). Aggressive surgical resection for hepatic metastases involving the inferior vena cava. Am. J. Surg..

[11] Kern P (2006). WHO classification of alveolar echinococcosis: principles and application. Parasitol. Int..

[12] Daniel D, Nicolas D, Pierre-Alain C (2004). Classification of surgical complications: a new proposal with evaluation in a cohort of 6336 patients and results of a survey. Ann. Surg..

[13] Chen KF (2018). The choose of different surgical therapies of hepatic alveolar echinococcosis: a single-center retrospective case-control study. Medicine.

[14] He YB (2015). Application of a three-dimensional reconstruction technique in liver autotransplantation for end-stage hepatic alveolar echinococcosis. J. Gastrointest. Surg..

[15] Vuitton DA (2016). Current interventional strategy for the treatment of hepatic alveolar echinococcosis. Expert. Rev. Anti Infect. Ther..

[16] Du L (2017). Combined resection of the right liver lobe and retrohepatic inferior vena cava to treat hepatic alveolar echinococcosis: a case report. Medicine.

[17] Hodjati H, Nazari SS, Nazhvani SD, Karami MY, Geramizadeh B (2017). Inferior vena cava reconstruction by gallbladder patch: an experimental design. Bull. Emerg. Trauma..

[18] Partensky C, Landraud R, Valette PJ, Bret P, Paliard P (1990). Radical and nonradical hepatic resection for alveolar echinococcosis: report of 18 cases. World J. Surg..

[19] Dziri C (1999). Omentoplasty in the prevention of deep abdominal complications after surgery for hydatid disease of the liver: a multicenter, prospective, randomized trial. French associations for surgical research. J. Am. Coll. Surg..

[20] Ijichi M (2000). Randomized trial of the usefulness of a bile leakage test during hepatic resection. Arch. Surg..

[21] Yoshidome H (2005). Should the inferior vena cava be reconstructed after resection for malignant tumors?. Am. J. Surg..

[22] Kieffer E, Alaoui M, Piette JC, Cacoub P, Chiche L (2006). Leiomyosarcoma of the inferior vena cava: experience in 22 cases. Ann. Surg..

[23] Malde DJ, Khan A, Prasad KR, Toogood GJ, Lodge JP (2011). Inferior vena cava resection with hepatectomy: challenging but justified. HPB.

[24] Brunetti E, Kern P, Vuitton DA (2010). Expert consensus for the diagnosis and treatment of cystic and alveolar echinococcosis in humans. Acta Tropica..

[25] Fattahi Masoom SH (2017). Albendazole therapy in human lung and liver hydatid cysts: a 13-year experience. Clin. Respir. J..

