# Author Correction: A new treatment strategy for end-stage hepatic alveolar echinococcosis: IVC resection without reconstruction

**DOI:** 10.1038/s41598-020-73529-8

**Published:** 2020-10-08

**Authors:** Qiancheng Du, Yanyan Wang, Mengzhao Zhang, Yichong Chen, Xuepeng Mei, Yanfei Li, Ying Zhou, Haining Fan

**Affiliations:** 1 Department of General Surgery, Shanghai Fourth People’s Hospital Affiliated to Tongji University School of Medicine, Shanghai, 200081 China; 2 The Graduate School, Qinghai University, Xining, 810000 China; 3 Department of Hematology, Affiliated Fuyang Hospital of Anhui Medical University, Fuyang, 236000 China; 4 Department of Hepatopancreatobiliary Surgery, The Affiliated Hospital of Qinghai University and Qinghai Province Key Laboratory of Hydatid Disease Research, Qinghai University, Xining, 81000 China

Correction to: *Scientific Reports* 10.1038/s41598-019-45968-5, published online 01 July 2019

This Article contains an error.

In the Results section, subheading ‘Patient follow up’, the Authors omitted relevant information about classification of the cause of death for the deceased patient. Even though the death of patient happened after 13 months after the surgery, the Authors were not able to identify any other possible cause of death and therefore this death was classified as operation-related.

Therefore, in this section

“There was 1 case of operation-related mortality because of upper gastrointestinal haemorrhage (Table 5).”

should read:

“There was 1 case of death (Table 5). Since no other possible cause of death was identified, this death was classified as operation-related mortality because of upper gastrointestinal haemorrhage.”

